# Editorial Note: Ectoparasite survey of schoolchildren in the Republic of Guinea

**DOI:** 10.1371/journal.pgph.0007167

**Published:** 2026-08-18

**Authors:** The PLOS Global Public Health Editors

The *PLOS Global Public Health* Editors issue this Editorial Note to inform readers that the work cited in this article [1] as Reference 32 was retracted before the article’s [1] publication date, and that the work cited as Reference 36 was retracted after the article’s [1] publication date. PLOS considers that these references are not crucial in supporting the research or conclusions reported in [1].

PLOS is aware that a large number of references listed in this article have had post-publication editorial action and PubPeer comments. These comments are linked to ethics and competing interest concerns, and PLOS does not consider that these concerns affect the scientific content of this body of supporting literature.
